# Average Rank-Based Score to Measure Deregulation of Molecular Pathway Gene Sets

**DOI:** 10.1371/journal.pone.0027579

**Published:** 2011-11-09

**Authors:** Huan Yang, Chao Cheng, Wei Zhang

**Affiliations:** 1 Department of Reproductive Endocrinology, Obstetrics and Gynecology Hospital, Fudan University, Shanghai, China; 2 Program in Computational Biology and Bioinformatics, Yale University, New Haven, Connecticut, United States of America; 3 Department of Molecular Biophysics and Biochemistry, Yale University, New Haven, Connecticut, United States of America; Broad Institute of Massachusetts Institute of Technology and Harvard University, United States of America

## Abstract

**Background:**

Deregulation of biological pathways has been shown to be involved in the turmorigenesis of a variety of cancers. The co-regulation of pathways in tumor and normal tissues has not been studied in a systematic manner.

**Results:**

In this study we propose a novel statistic named AR-score (average rank based score) to measure pathway activities based on microarray gene expression profiles. We calculate and compare the AR-scores of pathways in microarray datasets containing expression profiles for a wide range of cancer types as well as the corresponding normal tissues. We find that many pathways undergo significant activity changes in tumors with respect to normal tissues. AR-scores for a small subset of pathways are capable of distinguishing tumor from normal tissues or classifying tumor subtypes. In normal tissues many pathways are highly correlated in their activities, whereas their correlations reduce significantly in tumors and cancer cell lines. The co-expression of genes in the same pathways was also significantly perturbed in tumors.

**Conclusions:**

The co-regulation of genes in the same pathways and co-regulation of different pathways are significantly perturbed in tumors versus normal tissues. Our method provides a useful tool for better understanding the mechanistic changes in tumors, which can also be used for exploring other biological problems.

## Introduction

Cell behavior is under precise regulation by biological pathways, which consist of a series of biochemical reactions (metabolic pathways) or signal transduction events (regulatory pathways) [1], [2], [3], [4]. In normal cells, different pathways often act in a coordinated manner for regulating biological processes. However, in tumor cells, many important pathways are deregulated and cooperation between pathways is perturbed [5]. The involvement of specific pathways in tumorigenesis has been investigated intensively in previous studies [6], [7], [8], [9]. For example, the association of the mitogen-activated protein kinase (MAPK) signaling pathway with cancers has been reviewed in Wagner et al [10] and in Dhillon et al [11].

Microarray experiments provide the expression levels of tens of thousands of genes simultaneously, and have been widely used to understand the cancer mechanisms [12]. In recent years, interest has moved from single gene based analysis (e.g. identifying differentially expressed genes) to gene set based analysis [13], [14], [15]. The goal of most gene set based methods (more specifically, the pathway analysis) is to identify the cancer-associated pathways. This problem has been investigated in previous studies by examining the over-representation of pre-defined gene sets (pathways) in differentially expressed genes [16], or calculating more well-defined statistics based on gene expression profiles [17], [18], [19]. For instance, one of the most popular pathway analysis methods, called gene set enrichment analysis (GSEA) [19], calculates an enrichment score for each pathway. This score can be regard as a weighted Kolmogorov–Smirnov-like statistic and reflects the difference of the pathway between two sample groups (e.g. tumor vs normal). The statistical significance of the score is then estimated by using an empirical phenotype-based permutation test procedure. These methods are sensitive and powerful for identifying significant pathways associated with cancers. However, they cannot be used to explore the relationships between different pathways, which is also an important issue for cancer studies.

In this study, we calculated the normalized average rank of genes in pathways in a gene expression profile, denoted as AR-score (average rank based score), to represent the activity of the pathway. Similar rank-based statistics have been proposed in previous studies [20], [21], e.g. to understand microRNA regulation in breast cancer. Since the AR-score is rank based, it is robust to the systematic variance of samples (arising from inappropriate or incomplete normalization) in a microarray data set. AR-scores are thereby highly comparable between different pathways and samples. We applied the statistic to a large number of well-selected microarray expression data sets for normal tissues, different types of tumors and cell lines. We calculated the AR-scores of KEGG (Kyoto Encyclopedia of Genes and Genomes) pathways [22] in these samples and found that the resulting activity profiles are capable of distinguishing tumor/normal tissues or classifying different tumor subtypes. Moreover, we found that both the co-expression patterns of genes in the same pathway and the coordination between different pathways have been deregulated in tumor samples/cell-lines with respect to the normal tissues. In practice, the method we propose here can also be readily applied to other gene expression data for better understanding relevant biological problems.

## Results

### Calculation of AR-score for pathways

The human genome contains more than 23,000 protein-coding genes. Their protein products are organized into a complex network, in which they function coordinately to regulate many important biological processes. Specifically, the network is formed by a variety of intertwined biological pathways, which consist of a series of chemical reactions or signal transduction events. The activities of many pathways vary substantially among different tissues/cell-types, and are often subjects to considerable modifications in tumors with respect to the corresponding normal tissues.

We defined a new measurement called AR-score (AS) to quantify the activity of pathways in a biological sample based on its gene expression profile from microarray experiment. The AR-score for a pathway is calculated as the average rank of the relative expression levels of all genes in this pathway normalized by the total gene number. It takes a value from 0 to 1. A higher score indicates that genes in the pathway are overall highly expressed and therefore this pathway is highly active. The rank based score is robust by nature and can be directly used to compare pathway activities between different samples, even though they are not from the same microarray dataset. Moreover, the activities of pathways with different sizes (i.e. different in their gene numbers) are directly comparable.

### Pathway activities in human tissues

We calculated the AR-scores of 186 KEGG pathways in 79 human tissues for which the expression profiles were measured by microarrays [23]. For each of the pathways, we obtained an activity pattern, indicating the activities of the pathway across these tissues. We found that the activities of many pathways varied substantially between different tissues. For instance, the “CELL_CYCLE” pathway shows much higher activities in the tumor samples such as colorectal adenocarcinoma (AS = 0.65), B-lymphoblast (AS = 0.75) and leukemia promyelocytic hl60 cells (AS = 0.71) than in normal tissues. It also shows higher activity in the labile cells (cells that multiply constantly throughout life) such as CD71+ early erythroid bone marrow cells (AS = 0.74) than the quiescent cells such as cerebellum cells (AS = 0.35). Moreover, in fetal tissues the “CELL_CYCLE” pathway often shows higher activity than the corresponding adult tissues (e.g. AS = 0.65 in fetal liver, while AS = 0.38 in adult liver). These results were consistent with the functions of “Cell_Cycle” pathway genes in regulation of mitotic cell division: they are more highly expressed in tissue/cells with more active cell division.

**Figure 1:**
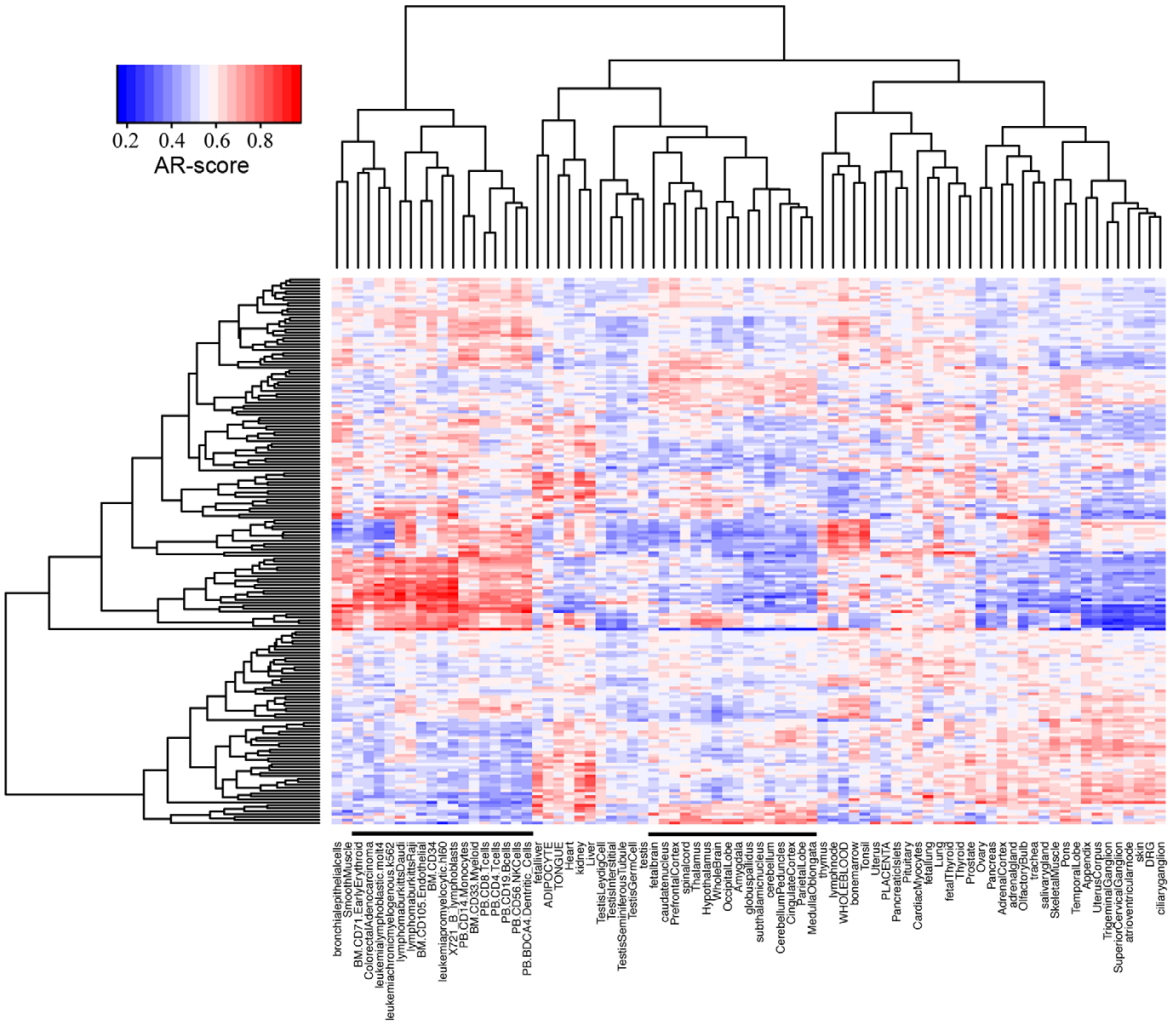
Hierarchical clustering of 79 human tissues based on their activity profiles. For each sample, the activity profile consists of the AR-scores for 186 KEGG pathways. The two lines mark the cancer cluster (left) and neuron cell cluster (right). The details are accessible in high-resolution images linked from the website.

On the other hand, the scores for the 186 KEGG pathways in a tissue give rise to an activity profile of the tissue. The pathway activity profile characterizes a tissue and can be used to investigate the similarity of different tissues in their metabolic/regulatory states. We performed the hierarchical clustering analysis for the 79 human tissues based on their pathway activity profiles. As shown in Figure 1, tissue/cells with relevant tissue origins are grouped into the same cluster (e.g. the blood cell cluster and the neuron cell cluster), suggesting that they have similar biological characteristics. Tumor tissues or cell lines form another cluster, indicating that they share common metabolic changes with respect to normal tissues (e.g. enhanced activity of “CELL_CYCLE” pathway).

### Coupling of different pathways in normal tissues

As the components of a whole network, different pathways often function in a coordinated manner to participate in many critical biological processes. To understand the inter-relationships among the KEGG pathways, we calculated the correlations of their activity profiles across the normal human tissues based on the dataset by Su et al [23]. We noted that many pathways overlapped in their gene members, which might lead to artificial positive correlations in their activity profiles. To overcome this problem, we excluded the shared genes between two pathways for their activity profile calculation and then calculated the correlation of the resulted activity profiles.

**Figure 2:**
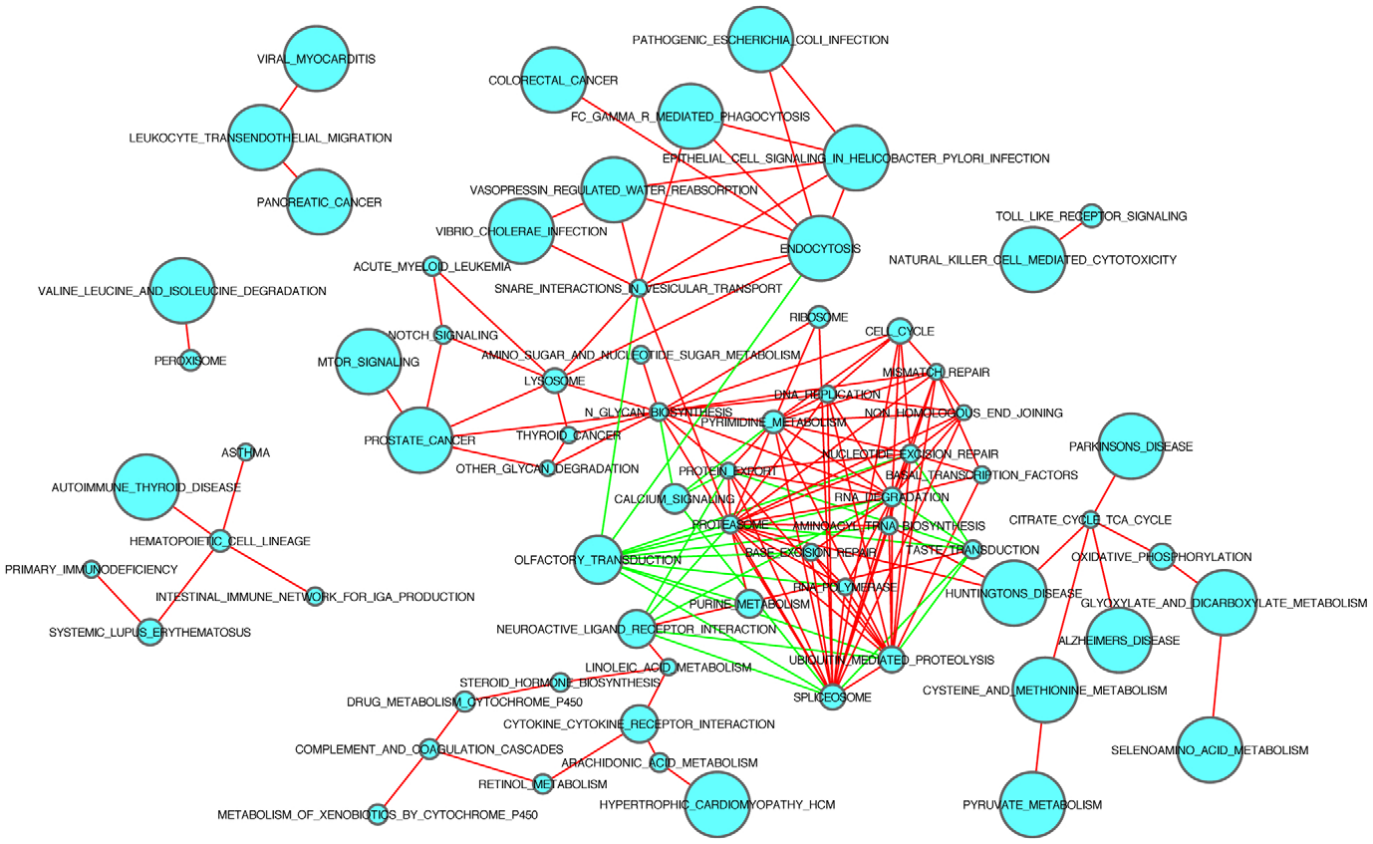
Correlation network of the KEGG pathways. Each node represents a KEGG pathway. The pathways that are positively (>0.75) or negatively (<−0.75) correlated are connected by "red" and "green" edges, respectively. The size of a node indicates the number of genes in the corresponding pathway. The details are accessible in high-resolution images linked from the website.

Our results indicated that the pathways were highly correlated in their activity in normal tissues. Among the 17,205 possible pathway pairs, 809 have a Spearman correlation coefficient ≥0.6 and 221 have a correlation ≤−0.6, corresponding to a P−value ≤10^−8^ (see Table S1 for all of the correlations). Figure 2 shows the correlation network of the 186 KEGG pathways using a more stringent cut-off value (|ρ|>0.75, P = 2×10^−14^). As shown, many pathways were positively correlated in their activity profiles (red edges), namely they are presumably coupled with each other. For example, the activity profile of the “CELL_CYCLE” pathway is positively correlated with those of the “DNA_REPLICATION”, the “MISMATCH_REPAIR”, the “NUCLEOTIDE_EXCISION_REPAIR” and 4 other KEGG pathways. More interestingly, we found that the “CITRATE_CYCLE_TCA_CYCLE” pathway was highly correlated with the “PARKINSONS_DISEASE”, “ALZHEIMERS_DISEASE” and “HUNTINGTONS_DISEASE” pathways. The high correlation of the pathway activity profiles indicated that different pathways were coupled with one another to achieve normal biological processes.

### Pathways with different AR-scores between normal tissues and tumors

**Figure 3:**
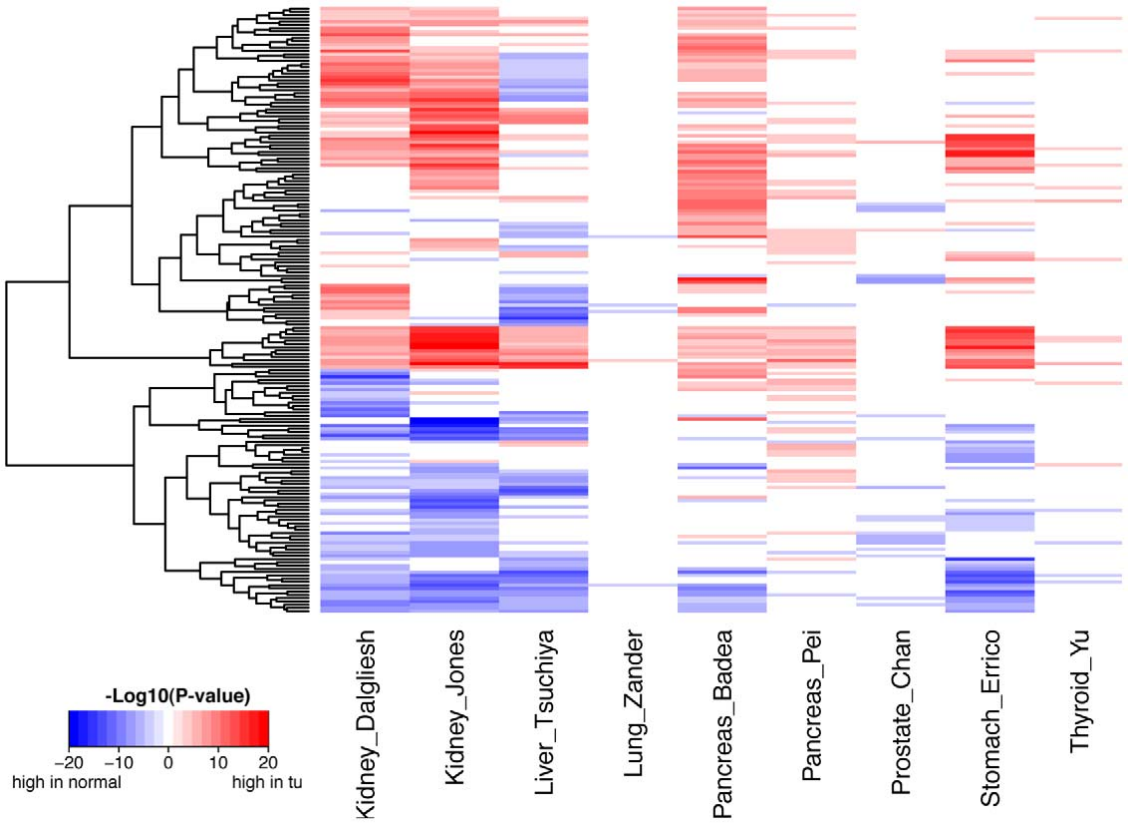
Pathways with differential activities between tumor samples and the corresponding normal tissues. The activity difference of a pathway in tumor and normal tissues was examined by the t−test and −log10(P−value) was color coded. Red indicates higher activity in tumors, and blue indicates higher activity in normal tissues. The results for 9 tumor/normal data sets are shown, representing 7 different cancer types.

To investigate the pathway modifications in tumor cells, we applied the pathway analysis to 9 cancer microarray data sets, representing 7 different cancer types: liver, lung, kidney, pancreas, prostate, stomach and thyroid cancers (see Table S2 for information about these data sets). For two of the cancer types (kidney and pancreas), we collected two independent data sets. These 9 data sets were carefully selected, each containing ≥15 tumor samples and ≥15 matched normal tissue samples. For each data set, we calculated the AR-scores of the 186 KEGG pathways in each of the samples, and then compared the AR-scores between the tumor samples and the normal tissues using the t−test. Figure 3 demonstrates the differential activities of these pathways in the 9 data sets, with red and blue representing pathways that show significantly higher activity in tumors and in normal tissues, respectively. For example, among the 186 KEGG pathways, 69 showed significantly higher activities in kidney cancer and 55 showed significantly higher activities in normal kidney samples (P<0.001) based on the “Kidney_Jones” data set. Thus, in kidney cancer the activities of a large fraction of the KEGG pathways (∼65%) are significantly affected, indicating substantial metabolic and regulatory modifications in tumor cells. We noted that the sensitivity of detecting pathway activity changes in cancer depends on the sample size of the microarray data sets. Thus, the number of differential pathways in the 9 data sets might not be directly comparable. In spite of this, the results were similar between different data sets for the same cancer type, e.g. the “Kidney_Dalgleish” and “Kidney_Jones”. Detailed information about differential pathways for each of the 9 data sets can be found in Table S3.

### AR-scores of pathways distinguish tumor and normal tissues

**Figure 4:**
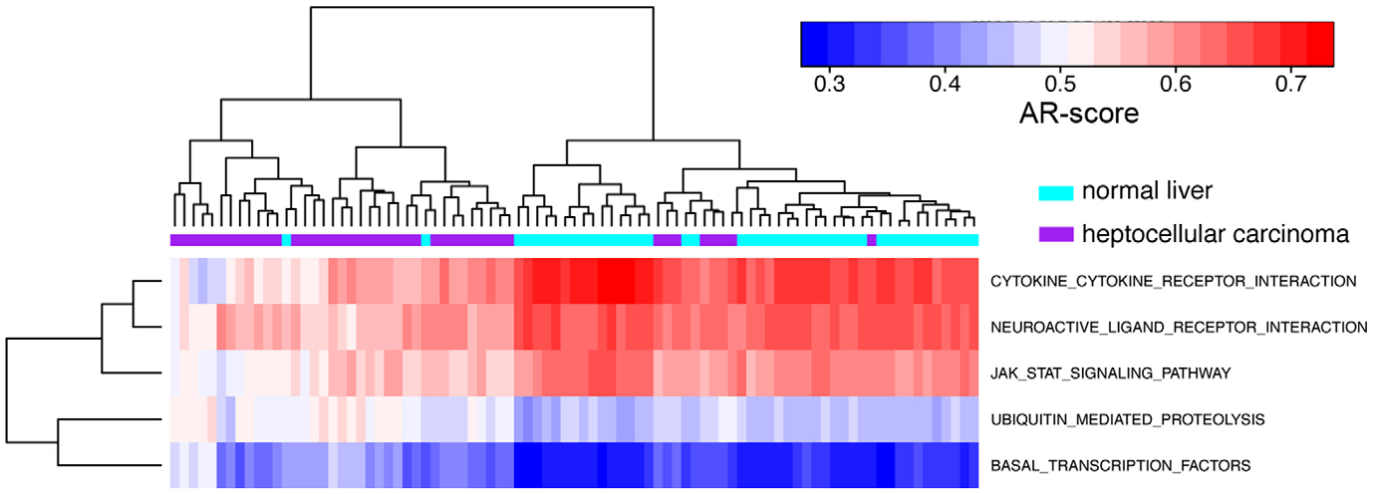
Hierarchical clustering of heptocellular carcinoma and normal liver samples based on the AR-scores for 5 pathways.

Given the substantial difference in the activities of many pathways between tumor and normal tissues, we then examined the effectiveness of classifying normal and tumor samples based on their activity profiles. Here we used the “Liver_Tsuchiya” data set as the example. The data set contained the expression profiles for 43 tumor and 44 non-tumor liver tissues surgically resected from patients with HCV-associated hepatocellular carcinoma. We established a support vector machine (SVM) to classify the tumor and non-tumor samples using the activity profiles for KEGG pathways as the classifiers, and examined the accuracy of the model based on the leave-one-out cross-validation method (see “Method and Materials” for details). When 62 pathways with significant activities between tumor and non-tumor samples (P<10^−5^) were used as the classifiers, the SVM model achieved 95% accuracy (83 out of the 87 samples). The same accuracy was obtained when the 11 most significant pathways were used (P<10^−10^). Impressively, a SVM model based on only 5 pathways (P<10^−12^) could still result in correct classification for 82 samples (94%). In contrast, the average classification accuracy was 0.85 when 5 randomly selected pathways were used as the classifiers. The classification power of these 5 pathways is also shown Figure 4: a simple hierarchical clustering analysis can roughly separate samples into tumor and normal groups. These results indicated that the activity profiles of KEGG pathways were highly informative for distinguishing tumor tissues from the normal tissues.

**Figure 5:**
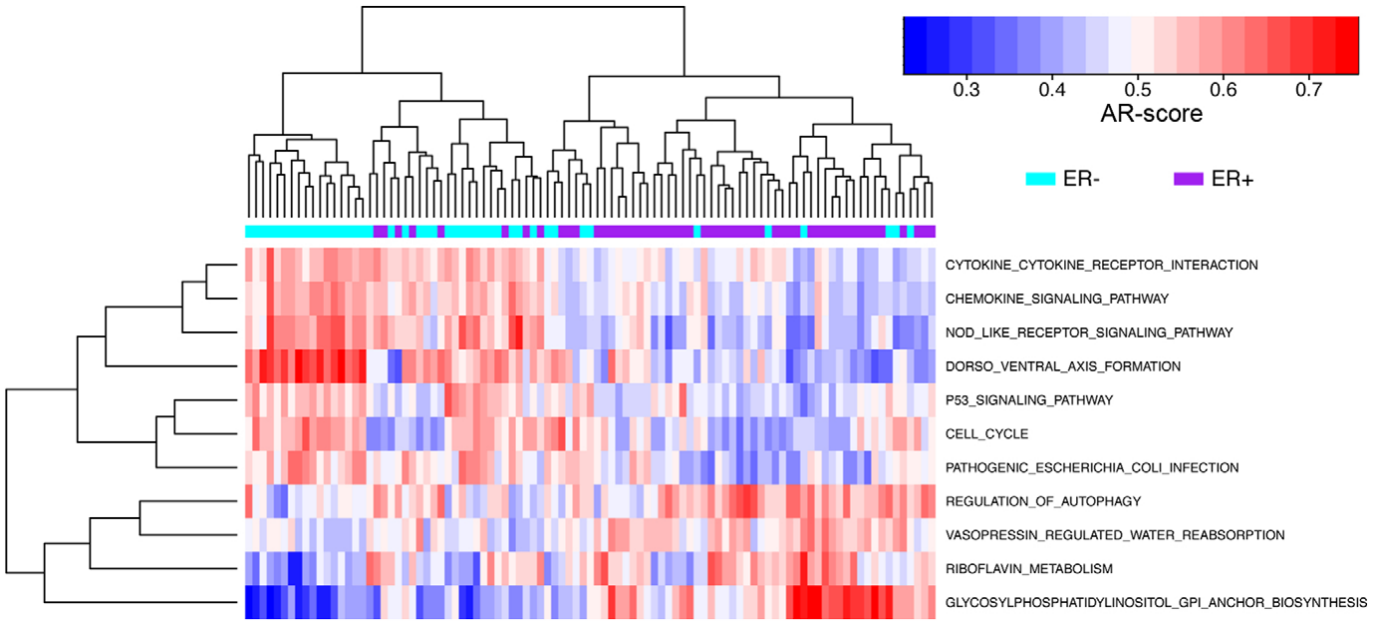
Hierarchical clustering of ER+ and ER- breast cancer samples based on the AR-scores for 11 pathways.

We then examined the effectiveness of classifying tumor subtypes based on the pathway activity profiles. As an example, we applied the SVM method to classify estrogen receptor positive (ER+) and negative (ER−) samples in the breast cancer data [24], which contained the expression profiles for 53 ER+ and 44 ER- breast cancer samples. The SVM method achieved 86% classification accuracy (83 out of 97 samples) when 65 most significant pathways (P<0.01) were used. Even when we reduced the number of pathways into 10 (P<10^−5^), the model still gave rise to a classification accuracy of 85% (83 out of 97 samples). The hierarchical clustering results based on these 10 pathways were shown in Figure 5. Thus, the activity profiles of pathways were capable of discriminating the subtypes of cancers.

We also examined the capability of the pathway activity profiles for predicting prognosis. In the breast cancer data from van’t Veer et al [24], the disease free survival (DFS) times for patients were available. We divided the patients into two groups based their DFS times: good-prognosis group (DFS≥60 month) and poor-prognosis group (DFS<60 month). We identified 5 pathways that showed significant activities between the two groups (P<0.001) and used them as the classifiers for the SVM model. Our model correctly classified 70 out of the 98 patients (71%). A logistic regression model based on expression levels of 70 genes instead achieves a prediction accuracy rate of 83% [24]. Despite the much decrease in classification accuracy, our results indicated that the pathway activity profiles were, to some extent, useful for predicting prognostic outcomes of cancer patients.

Gene-based classification of tumors has been intensively described in previous studies [24], [25]. Since the AR-score of a pathway summarizes the expression changes of all genes, we would expect more consistent results for different datasets of the same cancer type at the pathway level than at the gene level. Therefore, in comparison with genes, pathways are more stable classifiers for distinguish sample classes. For example, we examined the classification accuracies of 10 pathways and 20 genes in two independent kidney cancer data, the Dalgliesh data and the Jones data. These pathways and genes were the most differential ones between the cancer and the normal kidney samples in the Dalgliesh data [26]. In the same data, out of 160 samples a SVM model correctly classified 153 samples (96%) based on the 10 pathways, and 158 samples (99%) based on the 20 genes. In contrast, when applied to the Jones data, the pathway-based model still achieved high accuracy (86 out of 92 samples, 94%), whereas the gene-based model dropped to 90% (83 out of 92 samples).

### Reduced correlation of activity profiles between pathways in tumors

In normal cells, behavior is regulated by a number of pathways that cross-talk and are highly coordinated with each other. Cancer, in many ways, can be regarded as a disease of mis-regulated signal transduction. Thus, we would expect to see the decoupling of pathways in cancer with respect to normal cells. To investigate this issue, we carefully selected five microarray gene expression data sets, three for normal tissues (contain expression profiles for 73, 353 and 36 normal tissues, respectively) [23], [27], [28], one for cancers (contains 341 expression profiles for 6 different cancer types) [29], and one for NCI-60 cell lines (contains expression profiles for 60 tumor cell lines) [30]. These data sets represented a variety of normal tissues, tumor samples, or cell lines. In comparison with datasets containing only a single normal or tumor tissue type, these data sets carried more biological variations and therefore were more suitable for correlation analysis.

**Figure 6:**
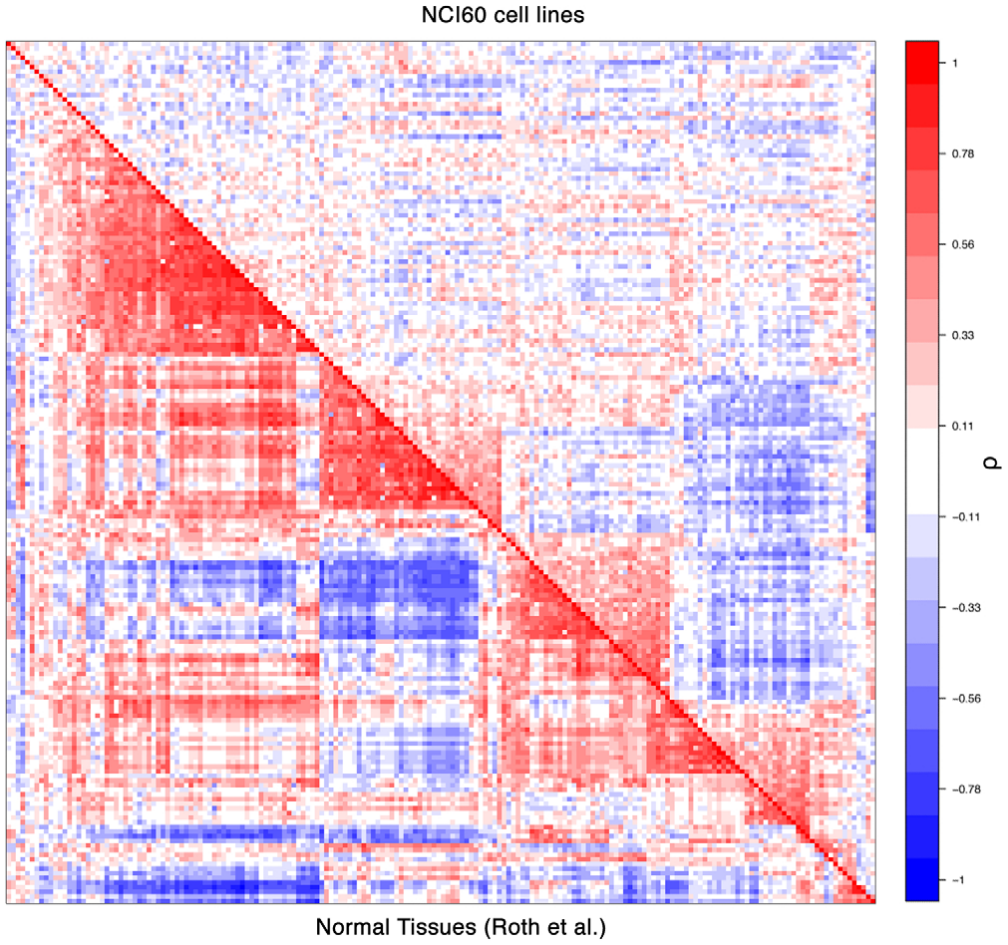
Reduced correlations of activities between pathways in cancer cell lines respect to normal tissues. The bottom left half shows the correlation matrix for 186 KEGG pathways in normal tissues. The top right half shows the correlation matrix for 186 KEGG pathways in NCI-60 cell lines.

We calculated the Spearman correlation coefficients for all pairs of the 186 KEGG pathways in the 5 data sets. Our results showed that the activity profiles of pathways were more correlated (either positively or negatively) in the normal data sets (Normal_Su, Normal_Roth, and Normal_Ge) than in the tumor and the NCI-60 data sets (NCI-60). Figure 6 shows the correlation patterns of the pathway activity profiles in Norml_Roth and the NCI-60 data sets (see Figure S1 for correlation patterns in all of the five data sets). Apparently, the pathways were decoupled in tumor samples and cell lines with respect to the normal tissues. Furthermore, the t-test showed that the correlation coefficients of the 17,205 pathway pairs in the normal data sets were significantly higher than those in the tumor data set and the NCI-60 cell line data: Normal_Su vs. tumor (t−score = 58), Normal_Su vs. NCI-60 (t−score = 61), Normal_Roth vs. tumor (t−score = 69), Normal_Roth vs. NCI-60 (t−score = 72), Normal_Ge vs. tumor (t−score = 45) and Normal_Ge vs. NCI-60 (t−score = 47), all corresponding to a very significant P−value (P<10^−308^). In addition, we examined the correlations of pathways in tumor and normal samples from the same dataset (see the 9 datasets described in Section “Pathways with different AR-scores between normal tissues and tumors”), which confirmed the conclusion that pathways were more correlated in normal tissues than in cancers. For example in the “Kidney_Dalgliesh” data, correlations (absolute values) between pathways are significantly higher in kidney cancers than in the normal kidney controls (t−score = 46, P<10^−308^).

### Reduced correlation of expression profiles for genes from the same pathway in tumors

**Figure 7:**
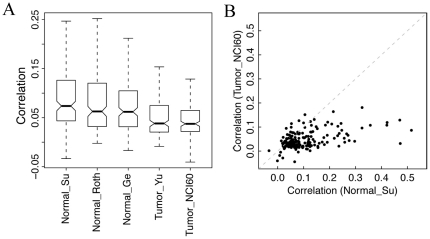
Reduced correlations of expression profiles for genes in the same pathways. For each of the 186 KEGG pathways, the average Spearman correlation coefficient was calculated across all pairs of genes in the pathway. (A) Distributions of the average correlations of the 186 pathways. (B) Average correlations of the 186 pathways in normal tissues and NCI-60 cell lines. Note most of the pathways show stronger gene co-expression in normal tissues than in cancer cell lines.

We have shown that the correlations of activity profiles between the pathways were reduced significantly in tumor samples or cell lines. We then asked: do the correlations of the expression profiles for genes in the same pathway also reduced in tumors? For each pathway we calculated the Spearman correlation coefficients for all gene pairs in the three normal data sets as well as in the tumor and NCI-60 data sets. The average of these pairwise correlations was then computed to represent the co-regulation of genes in a pathway. As shown in Figure 7, the expression profiles of genes in the same pathway were more correlated in the normal tissues than in the tumor and the NCI-60 data sets. For example, the average correlations of all gene pairs in the “RIBOSOME” pathway were 0.81, 0.76 and 0.72 in the three normal data sets, whereas the value was 0.41 in the tumor data set and 0.48 in the NCI-60 cell line data set (see Table S4 for the average correlations of all pathways). The reduced co-regulation of genes within the same pathway in tumors were further confirmed by comparing the average correlations of all the 186 pathways using the t−test: Normal_Su vs. tumor (P = 3×10^−11^), Normal_Su vs. NCI-60 (P = 5×10^−18^), Normal_Roth vs. tumor (P = 2×10^−13^), Normal_Roth vs. NCI-60 (P = 2×10^−17^), Normal_Ge vs. tumor (P = 2×10^−11^) and Normal_Ge vs. NCI-60 (P = 3×10^−15^).

### Comparison with the GSEA method

To further validate our results, we repeated the above-described analysis using the ES scores (enrichment scores) introduced in the GSEA method. Specifically, for each sample in a microarray data we calculated the ES-scores of all KEGG pathways. We compared the ES-scores of pathways in cancer versus normal samples to identify the differentially expressed pathways using the Wilcoxon test. The results we achieved are very consistent with those obtained from the AR-score based analysis (note that the t−test was used for comparing AR-scores). As an example, in the Liver cancer data we identified 88 significant pathways based on AR-scores and 85 significant pathways based on ES−scores (P<0.001). Among these pathways, 64 are identified by both methods. More importantly, among them the up-regulated and down-regulated pathways identified by the two methods are perfectly matched (see Table S5).

We also examined the correlation between different pathways in normal and cancer data sets based on their ES−scores. This analysis further validated our conclusion that the co-regulation of pathways tended to be decoupled in cancers with respect to normal tissues (see Figure S2). The correlation coefficients of ES−scores of pathways in the normal data sets were significantly higher than those in the tumor/cell-line data sets: Normal_Su vs. tumor (t−score = 32, P = 10^−208^), Normal_Su vs. NCI-60 (t−score = 39, P<10^−308^), Normal_Roth vs. tumor (t−score = 41, P<10^−308^), Normal_Roth vs. NCI-60 (t−score = 45, P<10^−308^), Normal_Ge vs. tumor (t−score = 28, P = 5×10^−163^) and Normal_Ge vs. NCI-60 (t−score = 33, P = 1×10^−226^).

## Discussion

Previous studies have reported the association of a number of pathways with cancers [8], [9], [11]. In fact, it has been shown that many oncogenes and tumor suppressor genes function as key factors in specific pathways [31], [32]. These studies, however, were centered mostly on the differential expression of individual genes or pathways. The co-expression of genes in the same pathways and the inter-relationships between different pathways have not been compared between tumor and normal tissues. In this study, we systematically investigated this issue using a large number of microarray data sets that contained expression profiles for normal tissues, different types of cancer tissues and cell lines. In cancer tissues and cell lines we observed the reduced correlation in expression profiles between genes from the same pathways as well as in activity profiles between different pathways. These results apparently suggested the connection of de-regulation of genes and pathways with carcinogenesis.

Several methods for pathway analysis have been previously proposed, such as Gene Set Enrichment Analysis (GSEA) [19]. These methods can be effectively used to detect pathways associated with cancer, but they are not designed for examining inter-relationships between pathways. The AR-score we propose captured the relative expression levels of genes in a pathway and can be used for understanding the co-regulation between pathways. As a rank based statistic, the AR-score is robust to noise and incomplete normalization of samples, and is highly comparable between pathways and samples. Despite of these advantages, it also has its limitations. When a pathway is active in a sample, we should not expect all proteins in the sample to be highly active. Only a fraction of active proteins may be active as a result of their functional redundancy. In a pathway, the proteins act either as activators or repressors. For repressors in an active pathway, we may not expect high expression of the corresponding genes. Instead, their expression may be down-regulated to activate a pathway. Moreover, the activities of some pathways may be largely regulated at the post-translational levels rather than at the mRNA level [33]. Thus the calculation of the AR-score can still be improved by taking these issues into account. In addition, it might also be improved by taking into account the variance of ranks of genes in a pathway, instead of simply averaging them.

The aforementioned limitations, however, are not likely to affect our main conclusions. Although genes in a pathway could be either up- or down- regulated in cancer versus normal, our results suggest that they tend to be regulated into the same direction. First, genes in the same pathway are more likely to be positively correlated in their expression profiles, which is particularly true in normal tissues (Figure 7). Second, for many pathways up- or down-regulated genes are predominant in the differentially expressed genes between tumor and normal. As an example, we counted the fraction of genes that were up-regulated in kidney cancer with respect to normal kidneys (Dalgliesh data), and found that in 56 out of 186 KEGG pathways ≥90% differentially expressed genes were actually up-regulated (Table S6). Third, the relative expression levels of genes were measured in Van’t Veer's breast cancer data (log ratios from the two-channel arrays), for which positive and negative values indicate up- and down-regulation, respectively. We examined the results of using a modified way to calculate the AR-scores of pathways. Namely, we ranked genes based on the “absolute” rather than the original values of their expression levels (log ratios). In this way, both the up- and down- regulated genes were taken into account, instead of canceling out with each other. Analysis based on the modified AR-scores again indicates perturbed co-expression of genes in the same pathway as well as perturbed co-regulation between different pathways in breast cancer with respect to normal tissues.

During human tumor development, the tumor cells have to obtain several capabilities, such as sustaining proliferative signaling and resisting cell death (Hanahan et al. 2001). Acquirement of these capabilities is often involved in the de-regulation of related pathways such as the apoptosis pathway. To de-regulate a pathway, the expression of a different subset of its genes might be perturbed in different tumor samples even for the same type of cancer. For this reason, the alteration of pathways is often more consistent than that of genes in tumor samples of the same type. For example, we compared the differentiation of pathways and genes between tumor and normal samples in two independent kidney cancer data sets. Specifically, the t-scores were calculated by comparing the AR-scores of pathways or expression levels of genes in tumors versus normal. The t-scores for pathways are more correlated (R = 0.81) between the two data sets than those for genes (R = 0.42), indicating high consistency between tumor samples at the pathway level. Such a consistency is only valid for those well-defined biological pathways, not for the random gene sets, which are mechanistically less informative. We examined the classification power of a number of random gene sets that are significantly different between tumor and normal samples. These random gene sets resulted in a classification accuracy (driven by several differentially expressed genes) that is only slightly lower than that of the real pathways. When applied to another data set, these gene sets are no longer effective for classification.

In brief, we propose a statistic to measure the pathway activities in gene expression profiles. We applied the method to a large number of microarray gene expression data sets to investigate changes of pathways in their activities and relationships in cancers with respect to normal tissues. We found that the AR-score we defined is capable of classifying samples into biological meaningful groups, e.g. normal vs tumor liver, and ER+ vs ER- breast cancer subgroups. We also found that in tumor samples and cell lines, the co-regulation of genes in the same pathways and between pathways was significantly perturbed with respect to normal tissues. More intensive investigation of the relationship changes between pathways would be helpful for further understanding the mechanisms of carcinogenesis.

## Materials and Methods

### Microarray gene expression data sets

A total of 14 microarray data sets were used in this study, among which three are for normal tissues, one is for cancer cell lines and ten are for tumor/normal samples. The breast cancer data was from van’t Veer et al [24], whereas the others were downloaded from the (GEO) database [34]. The accession IDs for normal tissues are GSE1133 by Su et al [23], GSE3526 by Roth et al [27], and GSE2361 by Ge et al [28]. The accession ID for the NCI-60 cell line data set is GSE5720 [30]. The accession IDs for the tumor/normal samples are GSE17895 and GSE15641 for kidney cancer [26], [35], GSE17856 for liver cancer [36], GSE12771 for lung cancer [36], GSE15471 and GSE16515 for pancreas cancer [37], [38], GSE6919 for prostate cancer [39], GSE13911 for stomach cancer [40], as well as GSE5364 for thyroid cancer [29]. All of these tumor/normal data sets contain gene expression profiles for ≥15 tumor samples and ≥15 corresponding normal tissue samples.

### Gene set information of pathways

The pathway information was downloaded from the database called Kyoto Encyclopedia of Genes and Genomes (KEGG) at http://www.genome.jp/kegg/ [22], where gene products were structured into 186 metabolic or signaling pathways.

### Preprocessing of Microarray data

Except for the breast cancer data from [24]and the liver cancer data from [36], the majority of gene expression data used in this work are performed using one-channel arrays. In these data, the absolute expression values of genes are measured. For each dataset, we performed gene-wise standardization by subtracting the mean and then dividing by the standard deviation of expression values of a gene in all samples. This step converts the expression of genes from absolute values into relative levels, which reflect their expressional variation in different samples. For the breast cancer and the liver cancer data, gene-wise standardization was not required, since gene expression in these two datasets was measured by two-channel arrays and represented originally as the relative values.

### Calculation of AR-score for pathways

Given the expression profile for a specific biological sample, we first sorted the expression levels for all genes in decreasing order. Then, we calculated the AR-score of a pathway by averaging the ranks of all genes in this pathway and then normalizing the result with the total gene numbers in the expression profile. Namely, 
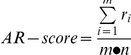
, where r_i_ is the rank of the i_th_ in the pathway, m is the number of genes in this pathway and n is total number of genes in the gene expression profile. The AR-score takes a value within (0,1), with a larger value indicating relatively higher expression levels of genes in a pathway and therefore a higher pathway activity.

### Correlation of activity profiles between pathways

The co-regulation of two pathways can be inferred based on the similarity of their activity profiles- their AR-scores in all of the samples. Simply, the Spearman Correlation Coefficient (ρ) between the activity profiles of two pathways can be used to measure the similarity. However, this would over-estimate the correlation if two pathways share common genes. To overcome this problem, we first removed genes shared by two pathways and calculated their AR-scores based on the remaining unique ones. The resulting AR-score profiles were subsequently used for estimating the correlation between the two pathways. Denote {x_i_, i = 1,2, …, p} and {y_i_, i = 1,2, …, p} as the rank vectors (p is the number of samples) for the AR-scores of two pathways, the co-regulation is calculated as



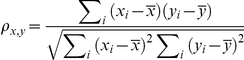
.

### Construction of correlation networks for pathways

We calculated the Spearman Correlation Coefficient for all possible pairs of the 186 KEGG pathways, while taking into account the shared genes between them (see Section “Correlation of activity profiles between pathways”). A total of 17,205 correlations were calculated, among which we observed much more positively correlated pathway pairs than negatively correlated ones. We then set |ρ|>0.75 as the cut-off value for correlations to select pathway pairs with similar AR-score profiles. Finally, all the pathways with |ρ|>0.75 were connected to form a co-regulation network for the pathways. The network contains both positive (correlated pathways) and negative (anti-correlated pathways) interactions. In normal tissues (Normal_Su), the above analysis resulted in a network with 59 nodes (pathways) and 127 edges (co-regulation relationships, |ρ|>0.75), among which 110 are positive correlations and 17 are negative correlations.

### Identification of differential pathways between different sample types

We used the t−test to identify the pathways that show differential activities between two sample groups (e.g. tumor versus normal). For instance, to identify pathways related to lung cancer, we compared the AR-scores of all pathways in lung tumor samples with those in normal lung tissues using the t−test, resulting a P−value for each pathway. To correct for multiple testing, we calculated the corresponding Q−value (false discovery rate, FDR) for each of the P−values using the method proposed by Storey et al [41]. The pathways with a Q−value<0.01 (1% FDR) were considered differential pathways between the two groups.

### Hierarchical clustering of samples based on pathway activity profiles

We performed hierarchical clustering to investigate the similarity of normal human tissues based on the AR-scores of the 186 KEGG pathways. Specifically, the “complete linkage” method was applied and the “Euclidian distance” was used as the dissimilarity metric in the hierarchical clustering analysis. Hierarchical clustering was also used to cluster tumor and normal samples or to cluster different tumor subtypes. For this purpose, we usually selected a number of pathways that showed differential activities and performed hierarchical clustering based only on these pathways. For example, Figure 5 was based on five pathways that are most significantly different between normal liver and heptocellular carcinoma.

### Classification of samples based on pathway activity profiles

We constructed support vector machine (SVM) models [42] to classify different sample types. For example, we used the SVM model to classify estrogen receptor positive (ER+) versus estrogen receptor negative (ER−) based on the AR-scores of the five most significant pathways. The classification accuracy was estimated by using leave-one-out cross-validation method. Each time a single sample was left out and the SVM mode was trained based on the remaining samples. The trained model was then used to predict the estrogen receptor (ER) status of the sample being left out. This procedure was repeated until each sample was left out once and we finally compared the predictions with the actual ER status to estimate the prediction accuracy of the classification model.

The SVM models were also used to classify normal versus tumor samples, and to predict the clinical outcome (good- or poor-prognosis groups) of patients.

### Correlation of expression profiles of genes in the same pathway

We calculated the Spearman correlation coefficient of the expression profiles for all possible pairs of genes in the same pathway. Then these correlations were averaged to represent the strength of co-expression of genes in this pathway. The average correlations were calculated for all of the 186 KEGG pathways. The average correlations for most pathways are positive values, particularly in normal tissues, indicating that genes in the same pathway tend to be co-expressed.

### Calculation of enrichment scores for pathways

To confirm our findings using the AR-score based method, we also performed all analysis using the statistic, “enrichment score” (ES), proposed by the GSEA method [19]. GSEA examines the distribution of genes in a gene set in a ranked gene list, which is sorted based on the correlations of genes with an interested phenotype. When applied to the case-control microarray data, GSEA is typically used to calculate the ES-scores for gene sets based on class comparison, e.g. the t-scores of genes in tumor versus normal. For comparison purposes, in this work we calculated the ES scores of the KEGG pathways in each relative expression profiles of a dataset, as we did for the AR-scores. The ES-score measures the maximum deviation between two cumulative distribution functions, and typically follows a bimodal distribution. In contrast, the AR-score is normalized average ranks of genes, which follows approximately a normal distribution (see Figure S3). Such a favorable feature of AR-score facilitates the subsequent downstream analysis, e.g. calculating correlation coefficient and comparing scores between sample classes using t−test, etc.

All the calculation described above was performed using the R language and packages available from http://www.r-project.org.

## Supporting Information

Figure S1 Correlation patterns of pathway activities (AR-scores) in five microarray data sets. The figure shows pairwise correlations of all pathways in three normal tissue data sets, one multiple-tumor data set and one NCI-60 cell line data set.
(PDF)

Figure S2 Correlation patterns of pathway activities (ES-scores) in five microarray data sets. The figure shows pairwise correlations of all pathways in three normal tissue data sets, one multiple-tumor data set and one NCI-60 cell line data set. Note that ES-scores are used to represent the pathway activities, whereas in Figure S1 AR-scores are used.
(PDF)

Figure S3 Distribution of AR-scores and ES-scores. Distributions of the AR-scores (left panel) and ES-scores (right panel) for pathways in Normal_Su data were shown. As shown, AR-scores approximately follow a normal distribution, while ES-scores follow a bimodal distribution with a positive and a negative peak.
(PDF)

Table S1 Correlations for all possible pairs of the KEGG pathways. Spearman correlation coefficients of all the 17,205 pairs of the 186 KEGG pathways were calculated.
(XLS)

Table S2 Information about the nine tumor vs normal data sets.
(XLS)

Table S3 Pathways showing differential activities between tumor and normal tissues in nine data sets. T-test was used to compare the activities of all the 186 KEGG pathways between tumor and normal samples in nine microarray data sets.
(XLS)

Table S4 Average correlations of expression profiles across all gene pairs in the same pathways. The table contains the average Sperman correlation coefficients for all the 186 KEGG pathways in three normal tissue data sets, one multiple-tumor data set and one NCI-60 cell line data set. For each pathway, the correlations for all possible pairs of genes in the pathway were calculated and averaged.
(XLS)

Table S5 Comparison of the differential pathways identified by the AR-score based and the ES-score based methods. The table contains the pathways that show significantly activities in Liver cancer and normal liver samples. The AR-scores of pathway in cancer and normal are compared using the t-test, whereas the ES-scores are compared using the Wilcoxon test. Note that pathways identified by the two methods are highly consistent.
(XLS)

Table S6 Percentage of up-regulated genes in pathways. The table contains the numbers of genes in each pathway, the fraction of up-regulated genes (t-score>0) in all genes or in the differentially expressed genes (P<0.001). Calculation is based on the Dalgliesh kidney cancer data.
(XLS)

## References

[1] Guarente L, Kenyon C (2000). Genetic pathways that regulate ageing in model organisms.. Nature.

[2] Mattson MP (2004). Pathways towards and away from Alzheimer's disease.. Nature.

[3] Rathmell JC, Thompson CB (2002). Pathways of apoptosis in lymphocyte development, homeostasis, and disease.. Cell.

[4] Rossi DJ, Jamieson CH, Weissman IL (2008). Stems cells and the pathways to aging and cancer.. Cell.

[5] Fritz V, Fajas L (2010). Metabolism and proliferation share common regulatory pathways in cancer cells.. Oncogene.

[6] Helleday T, Petermann E, Lundin C, Hodgson B, Sharma RA (2008). DNA repair pathways as targets for cancer therapy.. Nat Rev Cancer.

[7] Majumder PK, Sellers WR (2005). Akt-regulated pathways in prostate cancer.. Oncogene.

[8] Menashe I, Maeder D, Garcia-Closas M, Figueroa JD, Bhattacharjee S (2010). Pathway analysis of breast cancer genome-wide association study highlights three pathways and one canonical signaling cascade.. Cancer Res.

[9] Goc A, Al-Husein B, Kochuparambil ST, Liu J, Heston WW (2010). PI3 kinase integrates Akt and MAP kinase signaling pathways in the regulation of prostate cancer.. Int J Oncol.

[10] Wagner EF, Nebreda AR (2009). Signal integration by JNK and p38 MAPK pathways in cancer development.. Nat Rev Cancer.

[11] Dhillon AS, Hagan S, Rath O, Kolch W (2007). MAP kinase signalling pathways in cancer.. Oncogene.

[12] Russo G, Zegar C, Giordano A (2003). Advantages and limitations of microarray technology in human cancer.. Oncogene.

[13] Ackermann M, Strimmer K (2009). A general modular framework for gene set enrichment analysis.. BMC Bioinformatics.

[14] Liu Q, Dinu I, Adewale AJ, Potter JD, Yasui Y (2007). Comparative evaluation of gene-set analysis methods.. BMC Bioinformatics.

[15] Nam D, Kim SY (2008). Gene-set approach for expression pattern analysis.. Brief Bioinform.

[16] Pandey R, Guru RK, Mount DW (2004). Pathway Miner: extracting gene association networks from molecular pathways for predicting the biological significance of gene expression microarray data.. Bioinformatics.

[17] Tsai CA, Chen JJ (2009). Multivariate analysis of variance test for gene set analysis.. Bioinformatics.

[18] Oron AP, Jiang Z, Gentleman R (2008). Gene set enrichment analysis using linear models and diagnostics.. Bioinformatics.

[19] Subramanian A, Tamayo P, Mootha VK, Mukherjee S, Ebert BL (2005). Gene set enrichment analysis: a knowledge-based approach for interpreting genome-wide expression profiles.. Proc Natl Acad Sci U S A.

[20] Stuart JM, Segal E, Koller D, Kim SK (2003). A gene-coexpression network for global discovery of conserved genetic modules.. Science.

[21] Cheng C, Fu X, Alves P, Gerstein M (2009). mRNA expression profiles show differential regulatory effects of microRNAs between estrogen receptor-positive and estrogen receptor-negative breast cancer.. Genome Biol.

[22] Kanehisa M, Goto S (2000). KEGG: kyoto encyclopedia of genes and genomes.. Nucleic Acids Res.

[23] Su AI, Wiltshire T, Batalov S, Lapp H, Ching KA (2004). A gene atlas of the mouse and human protein-encoding transcriptomes.. Proc Natl Acad Sci U S A.

[24] van 't Veer LJ, Dai H, van de Vijver MJ, He YD, Hart AA (2002). Gene expression profiling predicts clinical outcome of breast cancer.. Nature.

[25] Dagliyan O, Uney-Yuksektepe F, Kavakli IH, Turkay M (2010). Optimization based tumor classification from microarray gene expression data.. PLoS One.

[26] Dalgliesh GL, Furge K, Greenman C, Chen L, Bignell G (2010). Systematic sequencing of renal carcinoma reveals inactivation of histone modifying genes.. Nature.

[27] Roth RB, Hevezi P, Lee J, Willhite D, Lechner SM (2006). Gene expression analyses reveal molecular relationships among 20 regions of the human CNS.. Neurogenetics.

[28] Ge X, Yamamoto S, Tsutsumi S, Midorikawa Y, Ihara S (2005). Interpreting expression profiles of cancers by genome-wide survey of breadth of expression in normal tissues.. Genomics.

[29] Yu K, Ganesan K, Tan LK, Laban M, Wu J (2008). A precisely regulated gene expression cassette potently modulates metastasis and survival in multiple solid cancers.. PLoS Genet.

[30] Shankavaram UT, Reinhold WC, Nishizuka S, Major S, Morita D (2007). Transcript and protein expression profiles of the NCI-60 cancer cell panel: an integromic microarray study.. Mol Cancer Ther.

[31] Croce CM (2008). Oncogenes and cancer.. N Engl J Med.

[32] Sherr CJ (2004). Principles of tumor suppression.. Cell.

[33] Wang X, Jiang X (2008). Post-translational regulation of PTEN.. Oncogene.

[34] Edgar R, Domrachev M, Lash AE (2002). Gene Expression Omnibus: NCBI gene expression and hybridization array data repository.. Nucleic Acids Res.

[35] Jones J, Otu H, Spentzos D, Kolia S, Inan M (2005). Gene signatures of progression and metastasis in renal cell cancer.. Clin Cancer Res.

[36] Tsuchiya M, Parker JS, Kono H, Matsuda M, Fujii H (2010). Gene expression in nontumoral liver tissue and recurrence-free survival in hepatitis C virus-positive hepatocellular carcinoma.. Mol Cancer.

[37] Badea L, Herlea V, Dima SO, Dumitrascu T, Popescu I (2008). Combined gene expression analysis of whole-tissue and microdissected pancreatic ductal adenocarcinoma identifies genes specifically overexpressed in tumor epithelia.. Hepatogastroenterology.

[38] Pei H, Li L, Fridley BL, Jenkins GD, Kalari KR (2009). FKBP51 affects cancer cell response to chemotherapy by negatively regulating Akt.. Cancer Cell.

[39] Chandran UR, Ma C, Dhir R, Bisceglia M, Lyons-Weiler M (2007). Gene expression profiles of prostate cancer reveal involvement of multiple molecular pathways in the metastatic process.. BMC Cancer.

[40] D'Errico M, de Rinaldis E, Blasi MF, Viti V, Falchetti M (2009). Genome-wide expression profile of sporadic gastric cancers with microsatellite instability.. Eur J Cancer.

[41] Storey JD, Xiao W, Leek JT, Tompkins RG, Davis RW (2005). Significance analysis of time course microarray experiments.. Proc Natl Acad Sci U S A.

[42] Liu HX, Zhang RS, Luan F, Yao XJ, Liu MC (2003). Diagnosing breast cancer based on support vector machines.. J Chem Inf Comput Sci.

