# Supplementary figures and images for: Average Rank-Based Score to Measure Deregulation of Molecular Pathway Gene Sets
Source: PLoS One. 2011 Nov 9;6(11):e27579. doi: 10.1371/journal.pone.0027579 (PMC3212578; doi:10.1371/journal.pone.0027579)

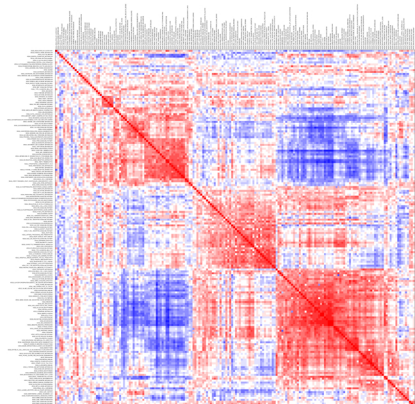

Normal (Su et al.)

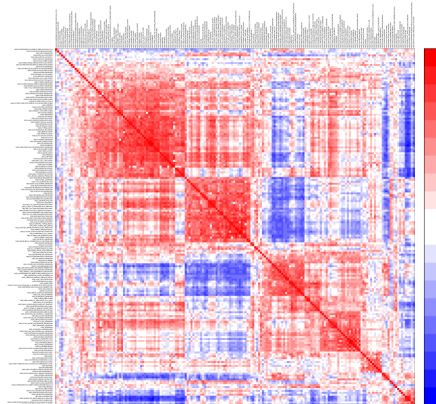

Normal (Roth et al.)

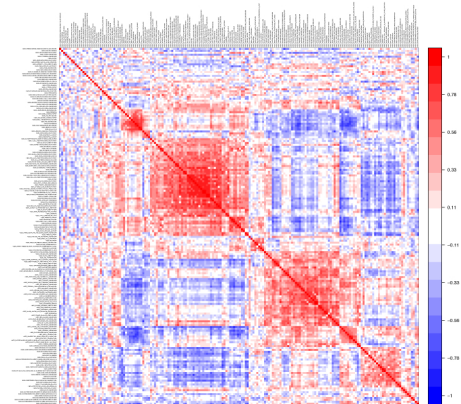

Normal (Ge et al.)

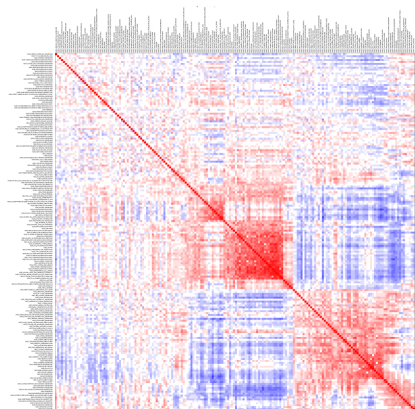

Tumor (Yu et al.)

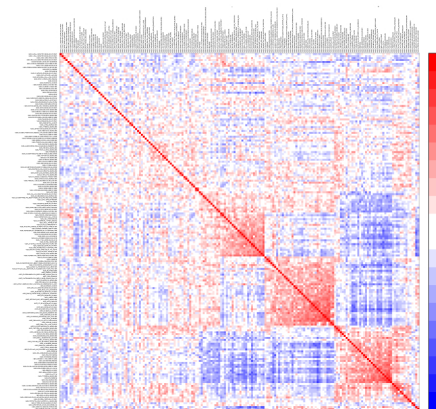

NCI60 cell lines (Shankavaram et al.)

Supplement: Figure S1 — Correlation patterns of pathway activities (AR-scores) in five microarray data sets. The figure shows pairwise correlations of all pathways in three normal tissue data sets, one multiple-tumor data set and one NCI-60 cell line data set. (PDF) [file pone.0027579.s001.pdf]

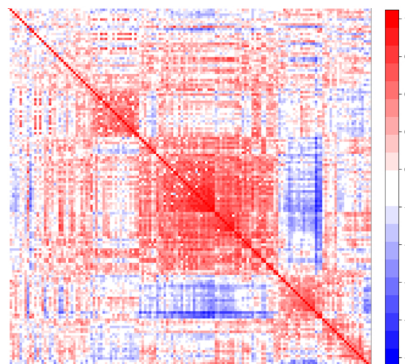

Normal (Su et al.)

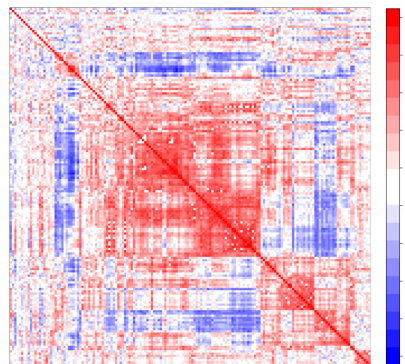

Normal (Roth et al.)

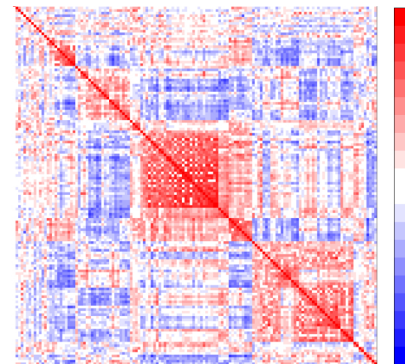

Normal (Ge et al.)

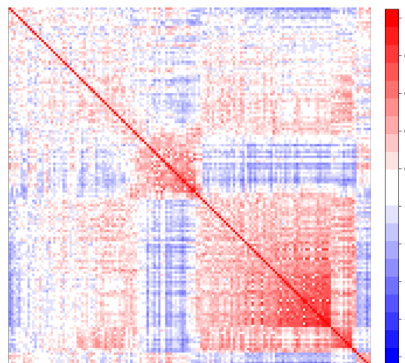

Tumor (Yu et al.)

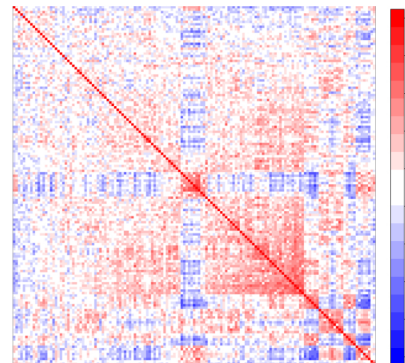

NCI60 cell lines (Shankavaram et al.)

Supplement: Figure S2 — Correlation patterns of pathway activities (ES-scores) in five microarray data sets. The figure shows pairwise correlations of all pathways in three normal tissue data sets, one multiple-tumor data set and one NCI-60 cell line data set. Note that ES-scores are used to represent the pathway activities, whereas in Figure S1 AR-scores are used. (PDF) [file pone.0027579.s002.pdf]

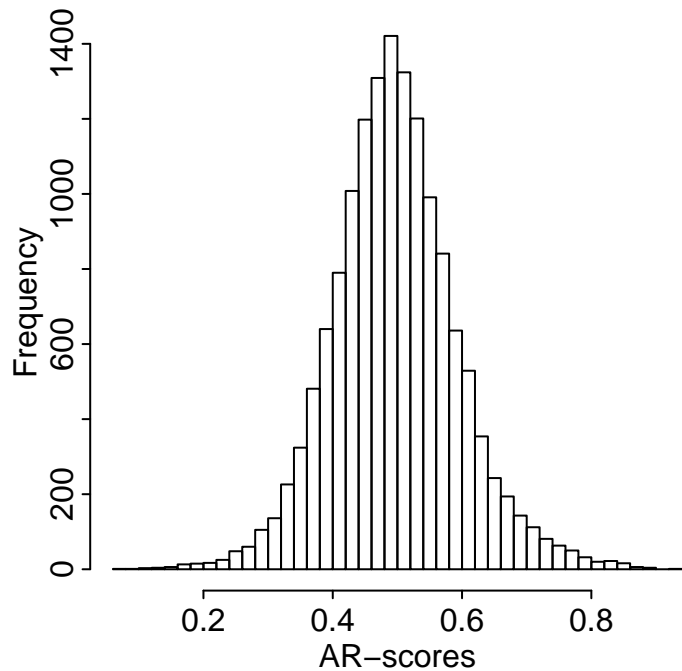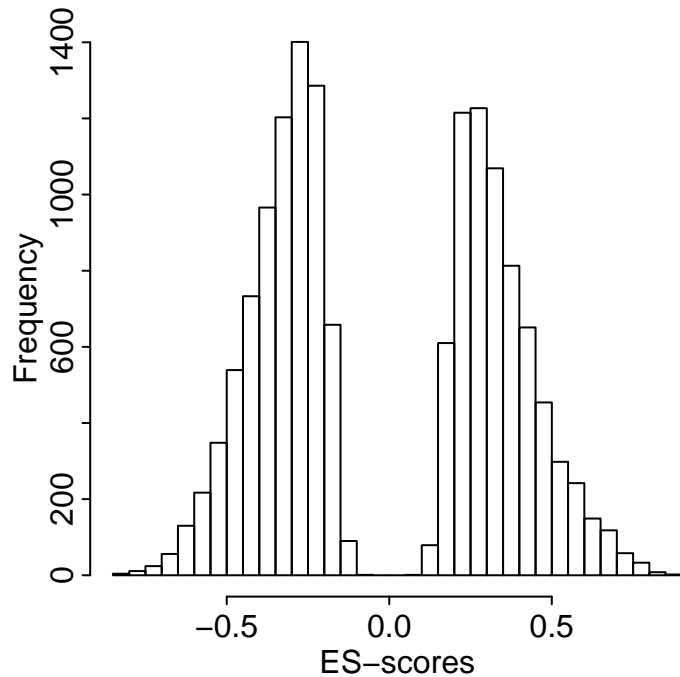

Supplement: Figure S3 — Distribution of AR-scores and ES-scores. Distributions of the AR-scores (left panel) and ES-scores (right panel) for pathways in Normal_Su data were shown. As shown, AR-scores approximately follow a normal distribution, while ES-scores follow a bimodal distribution with a positive and a negative peak. (PDF) [file pone.0027579.s003.pdf]
